# Supplementary material for: Ab Initio Molecular Dynamics Simulations of the Interaction between Organic Phosphates and Goethite
Source: Molecules. 2020 Dec 31;26(1):160. doi: 10.3390/molecules26010160 (PMC7795625; doi:10.3390/molecules26010160)
Supplement: Supplementary file 1 [file molecules-26-00160-s001.pdf]

Supplementary Information:

Ab Initio Molecular Dynamics Simulations of the Interaction between Organic Phosphates and Goethite

Prasanth B. Ganta,[a] Oliver Kühn,[a,b] and Ashour A. Ahmed,[a,b*]

[a]University of Rostock, Institute of Physics, Albert-Einstein-Str. 23-24, D-18059 Rostock, Germany

[b]University of Rostock, Department of Life, Light, and Matter (LLM), Albert-Einstein-Str. 25, D-18059 Rostock, Germany

* corresponding author

prasanth.ganta@uni-rostock.de

oliver.kuehn@uni-rostock.de

ashour.ahmed@uni-rostock.de

FIGURES

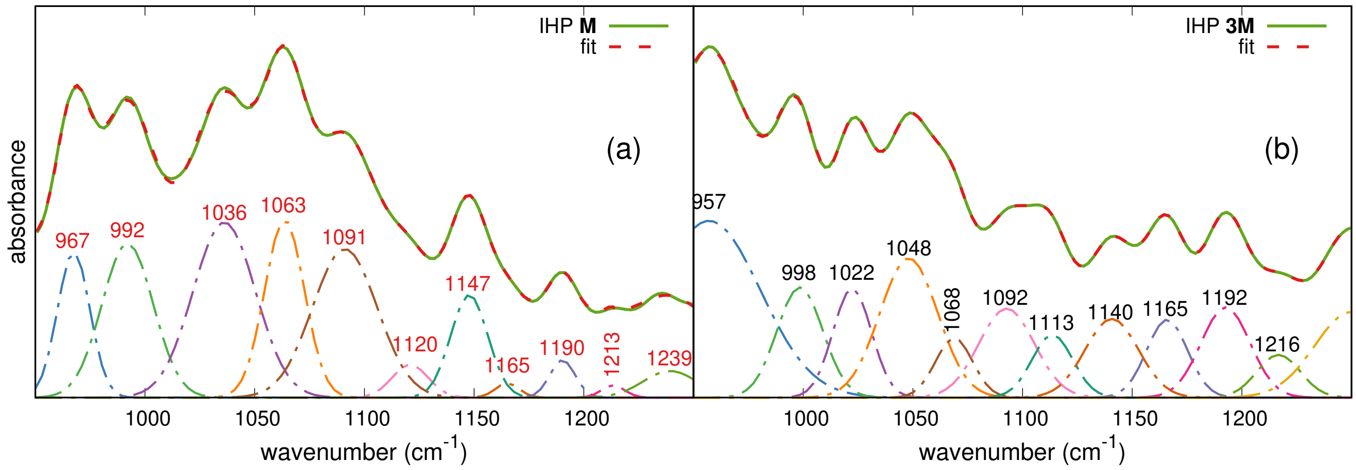

Figure S1: Fitted IR spectra of IHP bonded through **M** (a) and **3M** motifs (b).

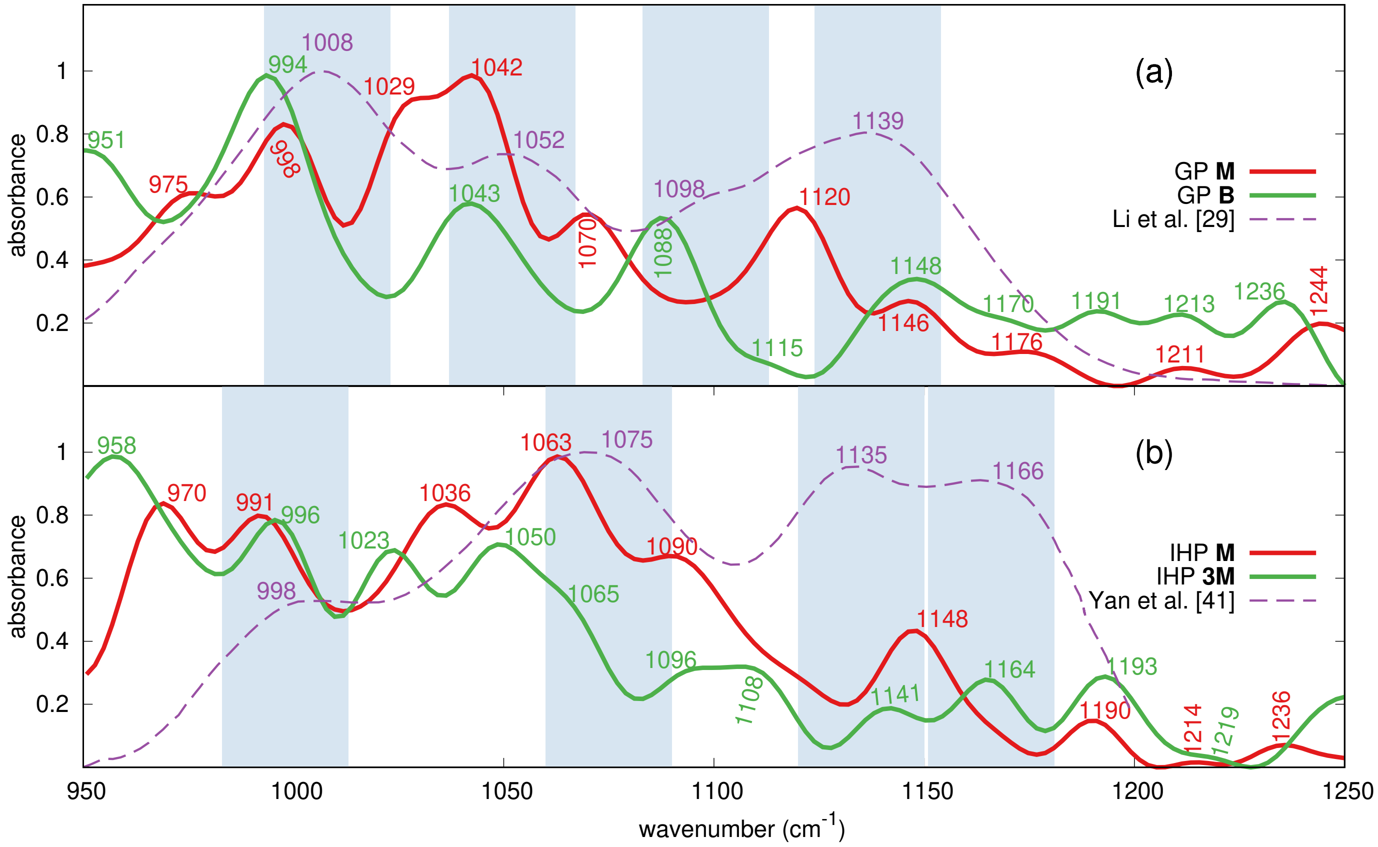

Figure S2: Comparision of GP and IHP spectra with experimental spectra from Li *et al.* [29] and Yan *et al.* [41] studies, respectively. The blue rectangle denotes region within ± 15 cm^{-1} around frequencies observed in experimental studies.

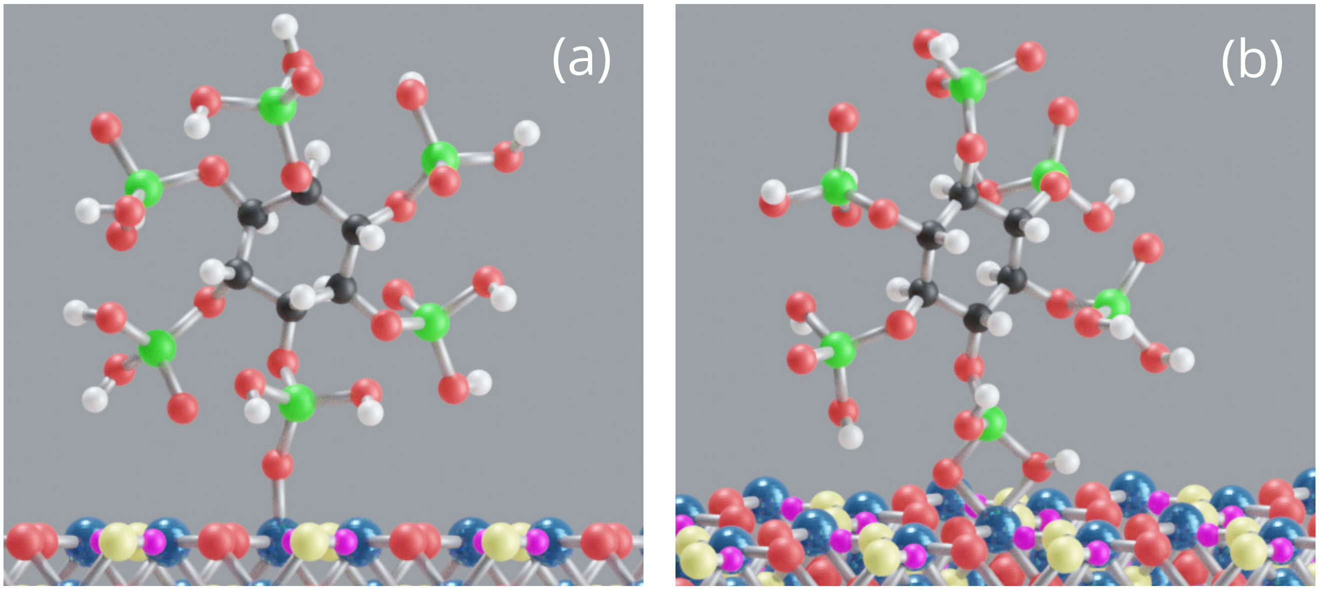

Figure S3: Initial motifs of IHP: **M** motif (a), **B** motif (b).

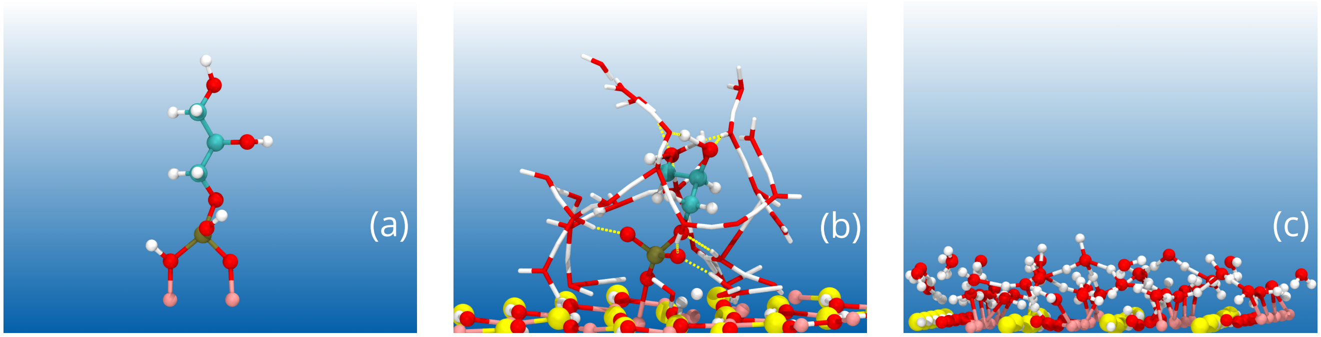

Figure S4: GP **BB** motif (a), HBs (yellow dotted lines) between GP and surrounding water (b), water bonded to surface Fe atoms (c). Pink, red, yellow, white and lime colors correspond to iron, oxygen, hydroxyl oxygen, hydrogen and phosphorus, respectively.

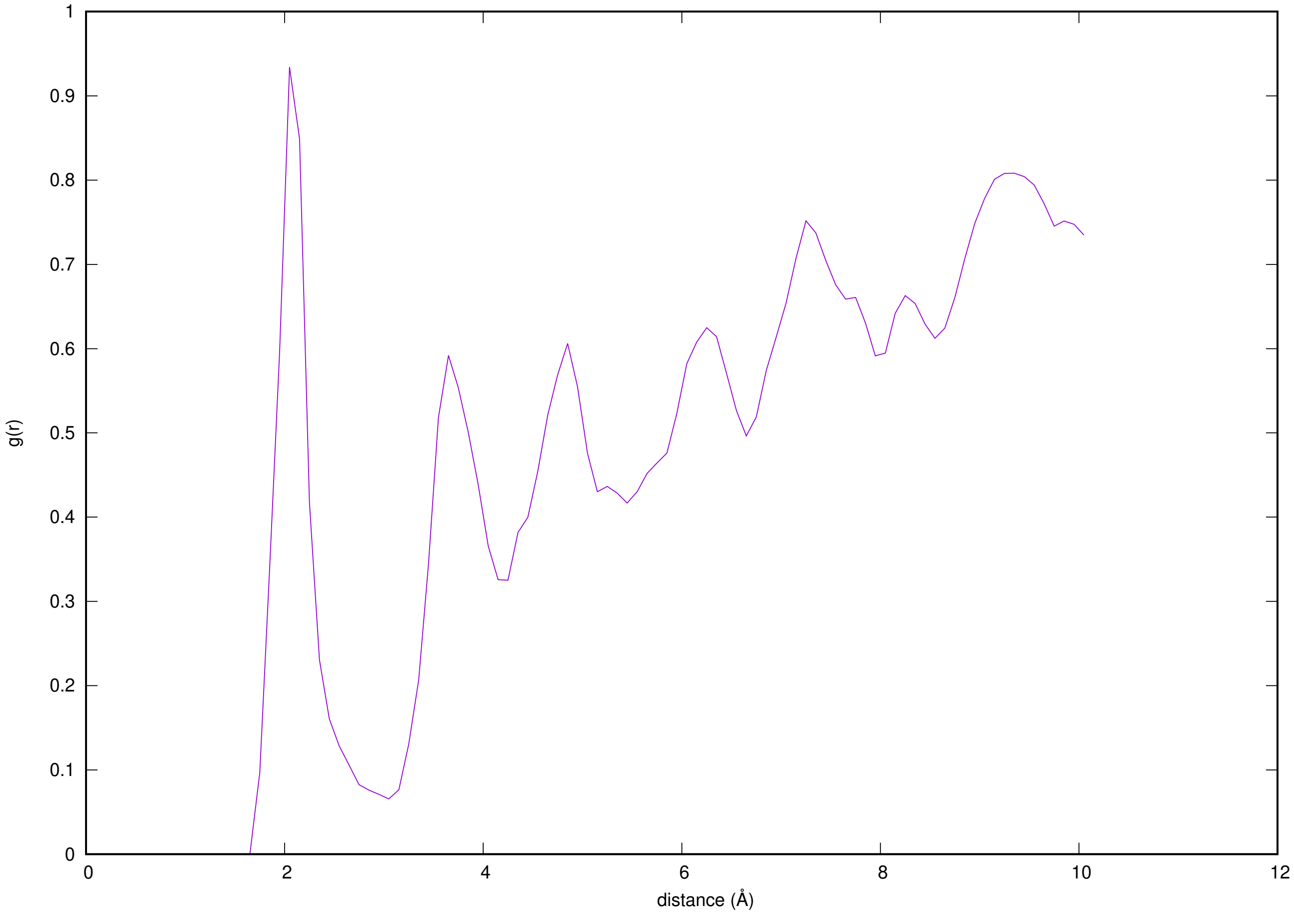

Figure S5: Pair correlation function calculated for surface Fe atoms of goethite and oxygens of water observed for GP **M** motif case.

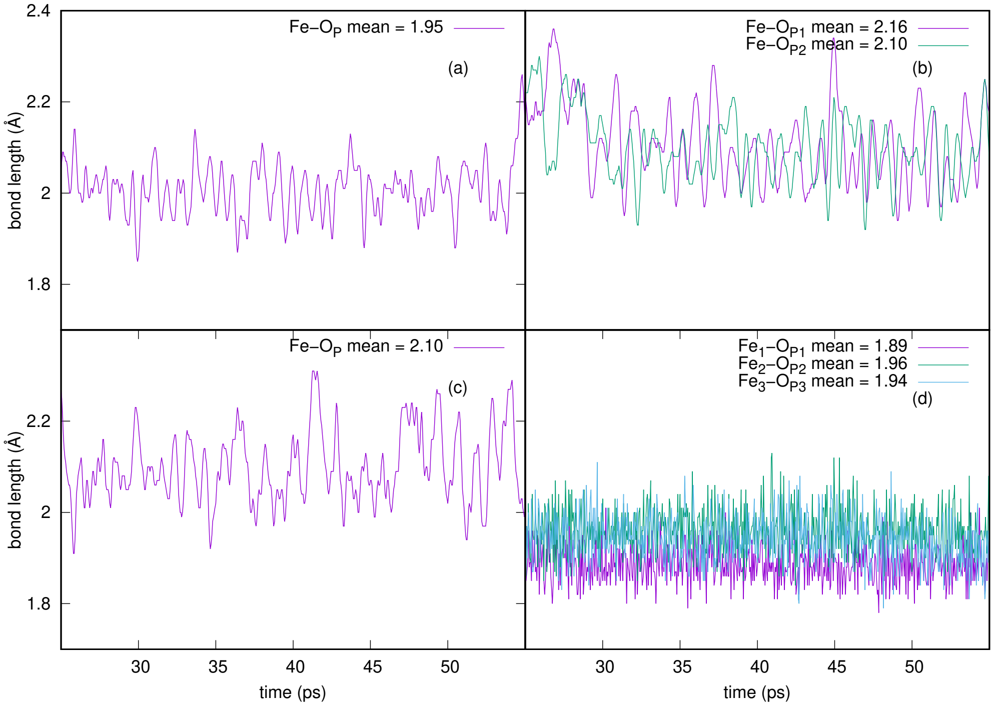

Figure S6: Covalent bond lengths of Fe-O$_P$ bonds for GP **M**(Fe–O$_P$) and **B**(Fe–O$_{P1}$, Fe–O$_{P2}$) motifs (a,b) and IHP **M**(Fe–O$_P$) and **3M**(Fe–O$_{P1}$, Fe–O$_{P2}$, Fe–O$_{P3}$) motifs (c,d) between 25–55 ps.

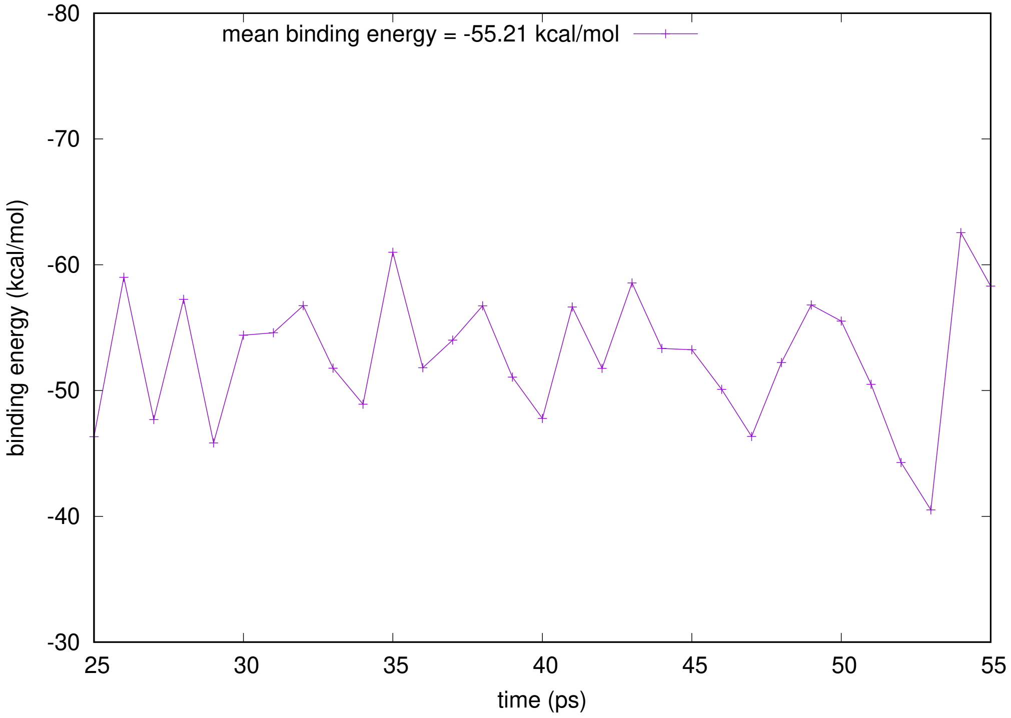

Figure S7: Binding energies along the simulation trajectory of IHP **M** motif in the time range of 25–50 ps.

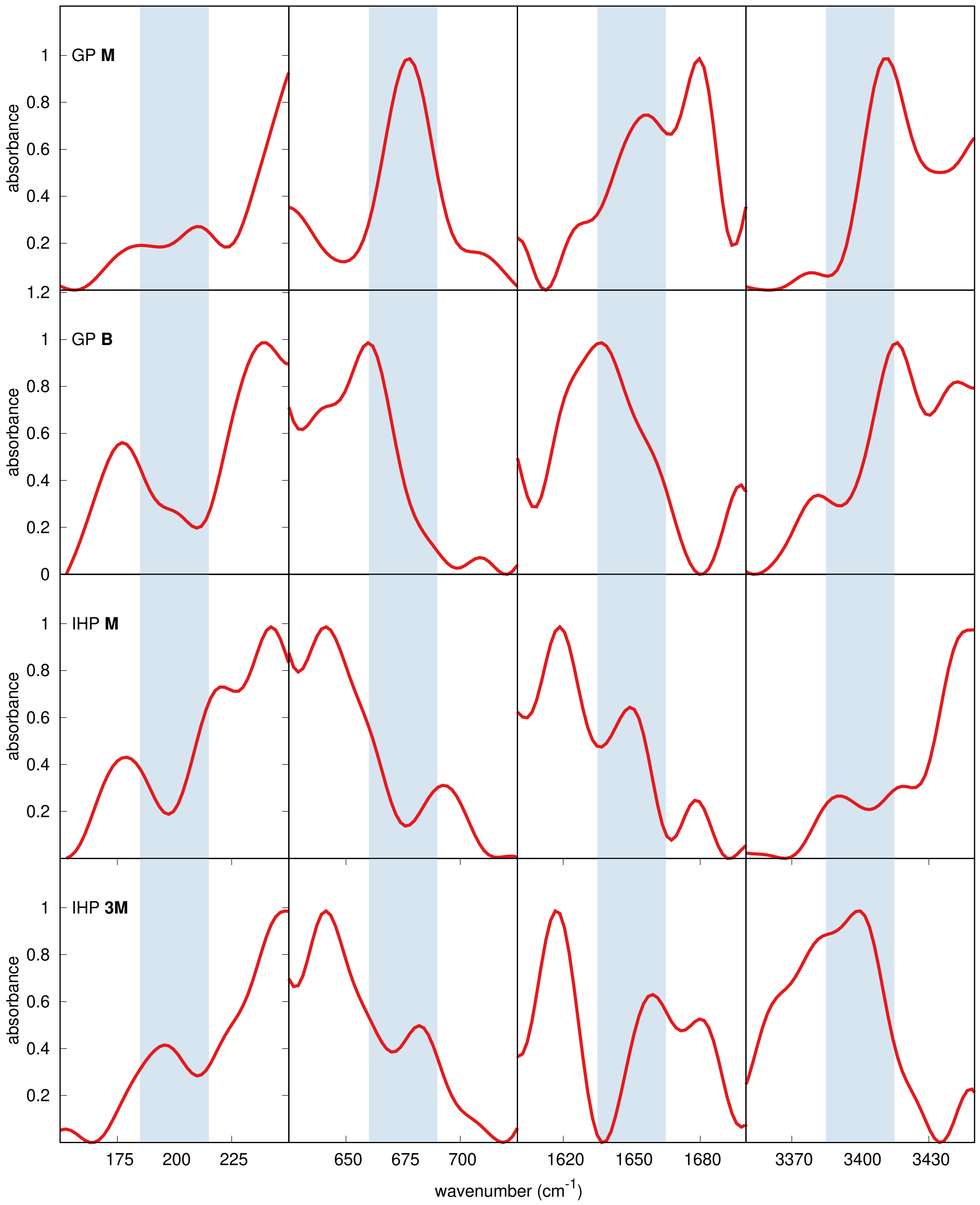

Figure S8: IR spectra of GP **M** and **B** motif cases (first and second rows) and IHP **M** and **3M** motif cases (third and fourth rows) for a selected freqency ranges. Brubach et al. [S1] showed that bands at 200, 675, 1650 and 3400 cm^{-1} are characteristic to water IR spectra and assigned the first two bands to hydrogen movement in intermolecular HBs and the last two to intramolecular [H–O–H] bending and [O–H] stretching modes. A similar assignment for [H–O–H] bending and [O–H] stretching modes is also observed in studies by Libnau et al. [S2] and Maréchal [S3]. The blue rectangle denotes a region within ±15 cm^{-1} around frequencies 200, 675, 1650 and 3400 cm^{-1}. The overall picture shows that peaks in selected freqency ranges of the calculated IR spectra match mostly with the peaks from the literature [S3, S2, S1].

References

[S1] J.-B. Brubach, A. Mermet, A. Filabozzi, A. Gerschel, P. Roy, Signatures of the hydrogen bonding in the infrared bands of water, J. Chem. Phys. 122 (2005) 184509.

[S2] F. O. Libnau, O. M. Kvalheim, A. A. Christy, J. Toft, Spectra of water in the near- and mid-infrared region, Vibrational Spectroscopy 7 (1994) 243–254.

[S3] Y. Maréchal, Infrared spectra of water. I. Effect of temperature and of H/D isotopic dilution, J. Chem. Phys. 95 (1991) 5565–5573.
